# David Julius and Ardem Patapoutian: Pioneers in Sensory Biology and Neuroscience

**DOI:** 10.7759/cureus.69779

**Published:** 2024-09-20

**Authors:** Charushila Rukadikar, Kadam Charulata, Satish P Dipankar, Milind A Nisargandha

**Affiliations:** 1 Physiology, All India Institute of Medical Sciences, Gorakhpur, Gorakhpur, IND; 2 Physiology, Symbiosis Medical College for Women (SMCW), Pune, IND; 3 Physiology, All India Institute of Medical Sciences, Mangalagiri, Mangalagiri, IND; 4 Physiology, Sundarlal Patwa Government Medical College, Mandsaur, Mandsaur, IND

**Keywords:** neuroscience, nociception, pain management, sensory biology, trpv1 receptor

## Abstract

Sensory biology is a critical area within neuroscience, exploring how organisms respond to environmental stimuli such as temperature, pain, and mechanical forces. David Julius and Ardem Patapoutian have made landmark contributions to this field by identifying crucial receptors. Julius's discovery of transient receptor potential (TRP) channels, including transient receptor potential cation channel subfamily V member 1 (TRPV1), which detects heat and pain, revolutionized sensory biology and opened new paths for pain management. Similarly, Patapoutian's identification of Piezo channels, which respond to mechanical stimuli like touch and pressure, has deepened our understanding of sensory perception. Their combined work has not only advanced scientific knowledge but also introduced potential treatments for chronic pain and sensory disorders. This paper reviews their contributions and the broader implications for sensory biology and therapeutic developments.

## Introduction and background

Sensory biology, a field critical to understanding how organisms perceive and interact with their environment, has been profoundly shaped by advancements in molecular biology and neuroscience. At its core, sensory biology studies how various stimuli such as temperature, pain, and mechanical forces are detected, processed, and interpreted by the nervous system. Understanding these mechanisms is fundamental to neuroscience and plays a pivotal role in developing novel therapeutic strategies for treating sensory-related disorders, such as chronic pain, inflammatory conditions, and neuropathies. A key breakthrough in sensory biology was the identification of specific receptors that detect external stimuli. These receptors, embedded in sensory neurons, serve as molecular gatekeepers, converting physical and chemical signals from the environment into electrical signals that the nervous system can interpret [1]. Among these, transient receptor potential (TRP) channels, particularly the transient receptor potential cation channel subfamily V member 1 (TRPV1), have garnered significant attention for their role in thermosensation and nociception (the sensory detection of harmful stimuli).

David Julius, a pioneering figure in this domain, made landmark discoveries that transformed our understanding of how the body perceives pain and temperature [2]. Julius’s identification of TRPV1, a receptor activated by capsaicin, the compound responsible for the heat sensation in chili peppers, unveiled the molecular mechanisms by which noxious heat is detected [3]. This discovery not only revolutionized sensory biology but also laid the foundation for developing new pain management strategies by targeting TRP channels [4].

**Figure 1:**
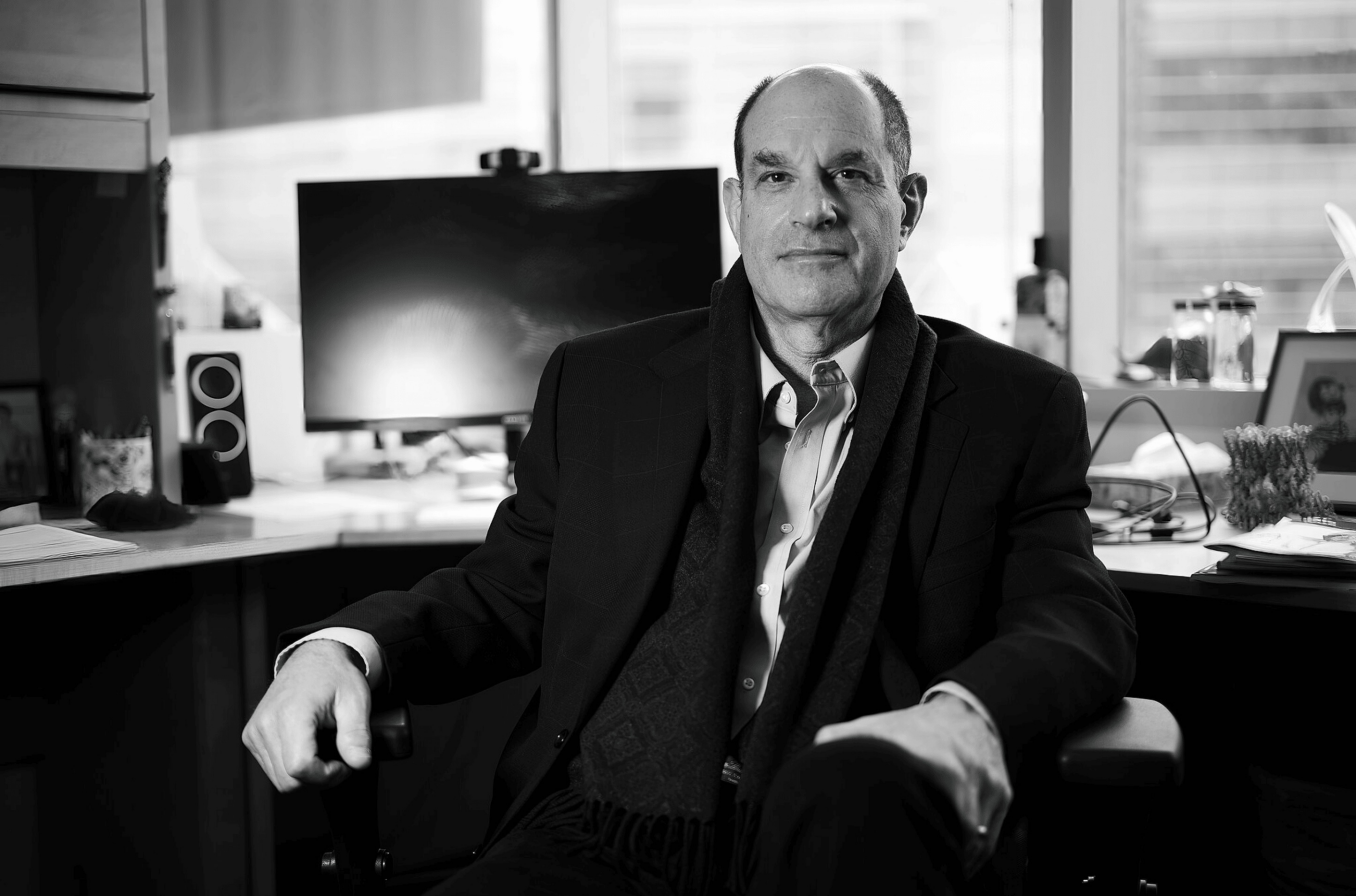
Dr. David Julius by Christopher Michel in 2022 This file is licensed under the Creative Commons Attribution-Share Alike 4.0 International license. Free to share, to copy, distribute, and transmit the work

Ardem Patapoutian made equally transformative contributions by focusing on mechanotransduction, the process by which cells detect mechanical forces. Using pressure-sensitive cells, Patapoutian discovered a novel class of sensors, Piezo1 and Piezo2 channels, that respond to mechanical stimuli such as touch and pressure. These discoveries have been crucial in understanding how the nervous system perceives mechanical force, allowing for a more complete understanding of sensory biology [5]. Together, Julius and Patapoutian’s work on TRP and Piezo channels has opened new therapeutic avenues, offering relief for millions suffering from chronic pain and sensory-related conditions [6,7].

**Figure 2:**
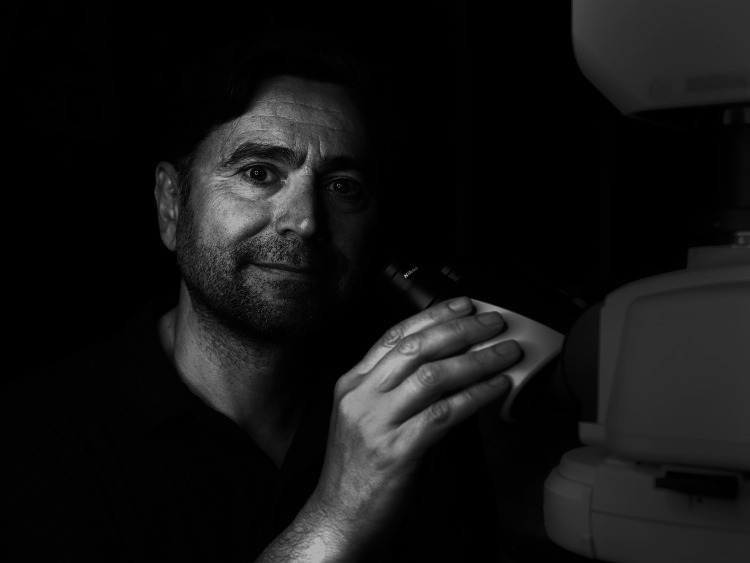
Ardem Patapoutian in 2022 by Christopher Michel This file is licensed under the Creative Commons Attribution-Share Alike 4.0 International license. Free to share, to copy, distribute, and transmit the work

In this article, we explore the pivotal role of TRP and Piezo channels in sensory biology, focusing on their molecular mechanisms, physiological functions, and potential as therapeutic targets. By analyzing the impact of Julius and Patapoutian's discoveries, we aim to provide a detailed understanding of how these channels contribute to sensory perception and how they may revolutionize the treatment of sensory-related disorders.

## Review

David Julius, born in 1955 in Brooklyn, New York, grew up in a household that fostered intellectual curiosity and a strong passion for scientific inquiry. His early exposure to stimulating academic environments led him to pursue higher education at one of the world’s most prestigious institutions, the Massachusetts Institute of Technology (MIT), where he earned his Bachelor's degree in Life Sciences in 1977. During his time at MIT, Julius was immersed in a rich environment that emphasized research innovation, which ignited his interest in molecular biology and sensory systems. After completing his undergraduate studies, he moved to the University of California, Berkeley, where he pursued his Ph.D. under the mentorship of renowned scientists Jeremy Thorner and Randy Schekman, two highly influential figures in the fields of yeast genetics and cellular biology. Julius completed his Ph.D. in 1984, focusing on the intricacies of cell biology and molecular mechanisms that govern sensory functions [7].

David Julius's career-defining breakthrough came when he identified the TRPV1 receptor, a key player in the TRP channel family. TRP channels are specialized proteins that reside in cell membranes and serve as molecular gatekeepers, allowing ions to flow into cells in response to various environmental stimuli. Julius’s identification of TRPV1 was a significant milestone in understanding how the human body detects and processes heat and pain. Using capsaicin, the active ingredient in chili peppers known for its heat-producing effects, Julius revealed how TRPV1 acts as a molecular sensor for noxious heat, providing critical insights into pain pathways. This discovery not only transformed sensory biology but also opened new doors in pain management research, particularly in developing therapies aimed at modulating TRP channels to alleviate chronic pain.

Building on his earlier success with TRPV1, Julius expanded his research to identify other critical receptors, namely, TRPM8 and TRPA1. TRPM8, which responds to cool temperatures and menthol, helped explain how the body detects cold sensations, while TRPA1 became known for its role in sensing chemical irritants, such as mustard oil, which produce a painful, burning sensation [8]. These findings deepened scientific understanding of the molecular mechanisms governing thermosensation (the sensing of temperature) and nociception (the perception of pain), advancing the broader field of sensory biology [9].

Julius’s contributions went beyond just identifying receptors. His research extended into understanding how toxins from venomous species interact with TRP channels, providing insights into how pain is modulated across different organisms. His work on toxins, such as those from tarantulas and snakes, further highlighted the evolutionary adaptation of pain receptors. Moreover, Julius’s use of cryo-electron microscopy (cryo-EM) enabled him to capture the precise structures of TRP channels at the atomic level, offering a detailed view of how these channels open and close in response to stimuli [10]. The impact of Julius’s work on pain biology cannot be overstated; his discoveries have laid the groundwork for novel pain-relief treatments that specifically target these sensory pathways, offering hope to millions of people suffering from chronic pain conditions [4].

Ardem Patapoutian, born in 1967 in Beirut, Lebanon, also made monumental contributions to sensory biology. Growing up during the Lebanese Civil War, Patapoutian experienced significant hardship, but his passion for science and discovery remained unwavering. In his early twenties, Patapoutian emigrated to the United States, where he pursued higher education at University of California, Los Angeles (UCLA) and later at Caltech. His academic journey in the United States honed his skills in molecular biology and neurobiology, eventually leading him to focus on mechanosensation, the process by which cells sense mechanical stimuli such as pressure, stretch, and touch.

In a groundbreaking series of experiments, Patapoutian identified PIEZO1 and PIEZO2 receptors, which are essential components of the body’s mechanotransduction pathways, mechanisms that allow cells to convert mechanical forces into electrical signals that the brain can interpret [11]. The discovery of these receptors has had profound implications for understanding how humans perceive touch, pressure, and proprioception (the sense of body position and movement). PIEZO2 was found to play a critical role in proprioception, helping the body maintain posture and coordinate movement. The role of PIEZO1 extended into vital physiological processes such as blood pressure regulation, immune response, and even cell division, showing that these channels are fundamental to various aspects of human health [12,13].

Patapoutian’s discoveries have provided valuable insights into conditions that affect sensory perception, including chronic pain, hypertension, and disorders related to mechanosensation. His work also opened new therapeutic avenues for treating sensory-related disorders, as targeting PIEZO channels could offer new approaches to managing conditions like mechanical allodynia, a painful condition where normally nonpainful stimuli, such as light touch, become painful. Patapoutian's research has also enhanced the understanding of mechanosensitive channels' role in broader biological systems, influencing research into inflammation and cancer [14].

Together, the discoveries of David Julius and Ardem Patapoutian in TRP and PIEZO channels have revolutionized sensory biology. Their work has bridged gaps in the understanding of how the body detects and responds to external stimuli such as temperature, pain, and mechanical forces. The impact of their research extends far beyond basic science, as their findings have laid the groundwork for innovative treatments in chronic pain management and other sensory dysfunctions. In 2021, Julius and Patapoutian were awarded the Nobel Prize in Physiology or Medicine for their pioneering discoveries, which continue to inspire new research directions and therapeutic possibilities. Their contributions have fundamentally altered how we approach the study of sensory systems, ensuring that their legacy in neuroscience and medicine will endure for generations [15].

## Conclusions

David Julius and Ardem Patapoutian's pioneering work in sensory biology and neuroscience has fundamentally transformed the understanding of how the human body detects and processes sensory stimuli such as temperature, pain, and mechanical forces. Julius's discoveries of TRP channels and Patapoutian's identification of PIEZO channels have revolutionized the study of sensory perception and opened new avenues for developing therapies for sensory disorders and chronic pain. Their research has laid the groundwork for innovative pain management strategies and has the potential to significantly improve the lives of individuals suffering from sensory dysfunctions. Their contributions continue to shape the future of neuroscience and therapeutic approaches in sensory biology.
